# An outbreak of *Salmonella Typhimurium* associated with the consumption of raw liver at an Eid al-Adha celebration in Wales (UK), July 2021

**DOI:** 10.1017/S0950268823001887

**Published:** 2023-11-30

**Authors:** James P. Adamson, Clare Sawyer, Gemma Hobson, Emily Clark, Laia Fina, Oghogho Orife, Robert Smith, Chris Williams, Harriet Hughes, Allyson Jones, Sarah Swaysland, Oluwaseun Somoye, Ryan Phillips, Junaid Iqbal, Israa Mohammed, George Karani, Daniel Rhys Thomas

**Affiliations:** 1 Communicable Disease Surveillance Centre, Public Health Wales, Cardiff, UK; 2 UK Field Epidemiology Training Programme, UK Health Security Agency, London, UK; 3 Microbiology, Public Health Wales, Cardiff, UK; 4 Communicable Disease, Health and Safety Team for Shared Regulatory Services for Bridgend, Cardiff & Vale of Glamorgan Councils, Cardiff, UK; 5 Lead for Service User Experience, Public Health Wales, Cardiff, UK; 6 School of Health Sciences, Cardiff Metropolitan University, Cardiff, UK

**Keywords:** *Salmonella Typhimurium*, Eid al-Adha, Whole genome sequencing, Lamb, Raw liver

## Abstract

In July 2021, Public Health Wales received two notifications of salmonella gastroenteritis. Both cases has attended the same barbecue to celebrate Eid al–Adha, two days earlier. Additional cases attending the same barbecue were found and an outbreak investigation was initiated. The barbecue was attended by a North African community’s social network. On same day, smaller lunches were held in three homes in the social network. Many people attended both a lunch and the barbecue. Cases were defined as someone with an epidemiological link to the barbecue and/or lunches with diarrhoea and/or vomiting with date of onset following these events. We undertook a cohort study of 36 people attending the barbecue and/or lunch, and a nested case-control study using Firth logistic regression. A communication campaign, sensitive towards different cultural practices, was developed in collaboration with the affected community. Consumption of a traditional raw liver dish, ‘marrara’, at the barbecue was the likely vehicle for infection (Firth logistic regression, aOR: 49.99, 95%CI 1.71–1461.54, *p* = 0.02). Meat and offal came from two local butchers (same supplier) and samples yielded identical whole genome sequences as cases. Future outbreak investigations should be relevant to the community affected by considering dishes beyond those found in routine questionnaires.

## Introduction

*Salmonella enterica* is a zoonotic, bacterial pathogen which is a common cause of gastrointestinal disease in humans [1], and is usually self-limiting. The most commonly reported serotypes of *Salmonella enterica* in the UK are *Salmonella Enteritidis* followed by *Salmonella Typhimurium* [2]. These two serotypes account for half of all salmonella laboratory reports in England and Wales [3]. Transmission of salmonella typically occurs through consumption of contaminated food or water, or through contact with animals and their environments [4]. Contamination of food and produce with salmonella can occur at any point as a result of poor hygienic practices during the production, storage, or preparation of food. In the UK, an estimated 39,000 cases (incidence = 582 cases per 100,000 population) of non-typhoidal salmonellosis occur in the community per year, of which around 10,000 are notified [5].

Foodborne outbreaks caused by salmonella in the UK are usually attributed to poultry and poultry products [6]. As a result, salmonella controls in livestock in the UK are targeted at poultry, and data on salmonella in poultry are collected as part of statutory surveillance systems [7]. Most salmonella reports from cattle, sheep, and pigs arise following investigation of diseased animals.

On a Friday in July 2021, Public Health Wales (PHW) Microbiology reported two cases of gastrointestinal disease with a positive PCR result for salmonella. Follow-up by Public Health Wales and Regulatory Services for Bridgend, Cardiff & Vale of Glamorgan Councils established that both cases were in individuals from the same Cardiff North African community who reported attending the same evening barbecue (from 6 pm) in a public park two days earlier to celebrate Eid al-Adha. It was also reported that celebration lunches for family and friends had also been held on the same day in three of the homes of this same social network (between 12 noon and 2 pm). Many people who attended one of the lunches were also present at single barbecue gathering the same evening. The barbecue was a ‘bring and share’ event served in a buffet style. The social network had purchased two whole lamb carcasses for the barbecue. This was bought cut (‘jointed’), ready to barbecue, from two local butchers who used the same supplier. This included cuts on the bone and offal (liver and kidneys) which cannot be guaranteed as being from the same animal as the flesh meat. The households hosting the lunches also prepared all the meat and offal dishes for the barbecue and some of vegetable and salad dishes. Meat for the lunches was bought from various local butchers, including those used to purchase the jointed carcases for the barbecue. No offal was served at the lunches, and meat at the lunches was stewed for several hours and served with boiled vegetables and salad. Other households in the same social network attending the barbecue prepared vegetable, salad, and sweet dishes for the barbecue, or bought sealed drinks, but did not buy or prepare meat or offal dishes. Meat cooked at the barbecue was served pink to well done. We initiated an epidemiological investigation in order to identify the source and extent of the outbreak, and to implement control measures to prevent further spread.

## Methods

### Case finding

Initial case finding was carried out by the Public Health Wales out-of-hours health protection service, in collaboration with local authority environmental health officers and Public Health Microbiology. We asked cases if they knew of other people who had become unwell after the barbecue and they reported there were friends and family in their social network who also had symptoms. We followed up these cases and at each conversation sought to uncover the extent of those who had become ill after attending the barbecue or a lunch event. For the purpose of the outbreak investigations, cases were defined as shown in Table 1.

**Table 1.** Case definitions used during the outbreak investigation

Case definition	Rationale
Possible outbreak case	Gastroenteritis case (diarrhoea and/or vomiting) with onset following the date of the lunches/barbecue, WITH an epidemiological link to the Eid al-Adha celebration barbecue or lunch
Probable outbreak case	Meets criteria for possible case AND has a PCR-positive result for *Salmonella Typhimurium* with or without a positive culture result **OR** Gastroenteritis case (diarrhoea and/or vomiting) with onset following the date of the lunches/barbecue, AND attended the Eid al-Adha celebration barbecue or a celebration lunch
Confirmed outbreak case	Meets criteria for a probable case and has a culture positive result for *Salmonella Typhimurium*
Cohort study case	An individual who attended the Eid al-Adha celebration barbecue and/or a celebration lunch, who became unwell with diarrhoea and/or vomiting within 8–72 h following the meal(s) attended and for whom a completed questionnaire was available
Whole genome sequenced case	An individual with *Salmonella Typhimurium* t5.4574.%(1.26.136.634.2037.4574.%)

All cases identified were interviewed, and risk of onward transmission was assessed. Standard enteric advice was given to cases and their contacts about infection prevention and control, and where necessary, cases were excluded from work or school. Information was collected about the personal characteristics of cases, their symptoms, food, and travel history, and a line list was maintained. All cases were asked to provide a stool sample for microbiological analysis.

### Cohort study

Those in the social network attending the celebration were included in a cohort study; attending the single evening barbecue and/or one of the celebration lunches. This was because attendees at lunches and/or the barbecue had the same exposure to meat purchased from the same butchers, either through preparation, consumption, or cross-contamination. The barbecue attendees were straightforward to define and verify. Lunch attendees were ascertained from trawling questionnaires and interviews which also determined who had prepared which dishes for the lunches and barbecue. The names and household residence of attendees of the lunches and barbecue were cross-referenced and verified by discussing who was present at which meal, with cases and other members of their household. We tested the hypothesis that being a case was associated with consumption of sheep meat/offal products served at the celebration meals. We designed and administered a questionnaire survey to all those attending a celebration lunch and/or the evening barbecue. This was based on feedback from several sources about the food served at these various meals. This questionnaire was administered by face-to-face field interviews (as the preferred option) to individuals or a family unit as participants desired, telephone interview (second preference), or by the online questionnaire tool ‘SmartSurvey’ [8]. The majority of interviews were conducted in English, but for one telephone interview, an Arabic interpreter was used by utilising the Language Line service [9]. We asked about details of the specific dishes consumed and the portion size of each. We asked and clarified who had prepared each dish served at the lunches and the barbecue. The details of ingredients and preparation were checked with those who prepared the dishes. Information on demographics, clinical features, outcomes, and exposures was collected, as was travel history, contact with animals, and contact with infectious individuals in the 48 hours prior to the day of the celebration meals.

### Descriptive epidemiology and statistical analysis

We described the characteristics of cases and their exposure history, and constructed an epidemic curve. Using our cohort of people attending the barbecue and/or celebration lunch, we compared the likelihood of being a case in those consuming specific food items. Analysis was undertaken using STATA 15 to calculate p-values using Fisher’s exact test due to sample size. An R package was used [10] to calculate 95% confidence intervals through the Wilson score method [11] because it is suited to small samples and likely to avoid the situation where p-values and confidence intervals ‘disagree’ if a Wald normal approximation is used.

We examined whether those who ate more of an implicated food were more likely to have severe illness. This dose–response analysis was possible from asking survey respondents how much of each food they ate (a small, normal or large portion). Severity of disease was assessed by using their responses about seeking medical care. Those attending hospital were considered more ill than those who did not (no cases contacted their GP) and those who were admitted to hospital were considered most ill of all.

Further analysis was carried out using a nested analysis and Firth logistic regression to investigate whether lamb or raw offal consumption was important. Firth regression is based on case–control design (odds ratios), and we used the same cohort for this analysis. This analysis was used to allow for zero-cell counts or unanimous consumption of certain food types and further examine lamb consumption in particular. We assessed the same exposures as the cohort study (foods consumed) and controls were defined as people attending the barbecue and/or a lunch event who did not become ill. Significant odds ratios (*P* < 0.05) calculated in the univariate results were included in the Firth regression, and interactions for age and sex were also tested.

### Microbiology and whole genome sequencing of human samples

Stool samples were sent to Public Health Wales Microbiology, Cardiff, for PCR testing. Those returning a positive PCR result for one of the standard multiplex panel pathogens (5 bacteria, 1 virus, and 2 parasites: Salmonella, Shigella, Campylobacter, STEC, *C. difficile*, Norovirus, Cryptosporidium, and Giardia) were cultured and subtyped locally. Samples of positive cultures were also sent to the UK reference laboratory (Gastro Bacterial Reference Unit (GBRU) in Colindale, UK Health Security Agency (UKHSA)) for whole genome sequencing (WGS). Full single nucleotide polymorphism (SNP) addresses are reported by this service, with the 5 SNP level used for cluster designation and public health action.

### Food microbiology

Households containing a case were asked to provide samples of any uncooked, unused, or leftover food items from the barbecue or lunches. Environmental and food samples were also taken from the two local butchers and supplying abattoir. These samples were sent initially to Public Health Wales’s Food, Water and Environment (FEW) laboratories (University Hospital Llandough, Cardiff) and tested for salmonella only using culture (horizontal method for the detection; BS EN ISO 6579-1:2017 + A1:2020 [12]) for enumeration and serotyping, before referring isolates to GBRU for whole genome sequencing. Samples were tested for salmonella only due to the cost and time associated with multiple tests and because *Salmonella Typhimurium* had already been detected in human stool samples from cases in this outbreak by the time of food sample testing. There were also ongoing capacity issues in laboratories in Wales due to the COVID-19 pandemic response.

## Results

### The outbreak

**Figure 1.**
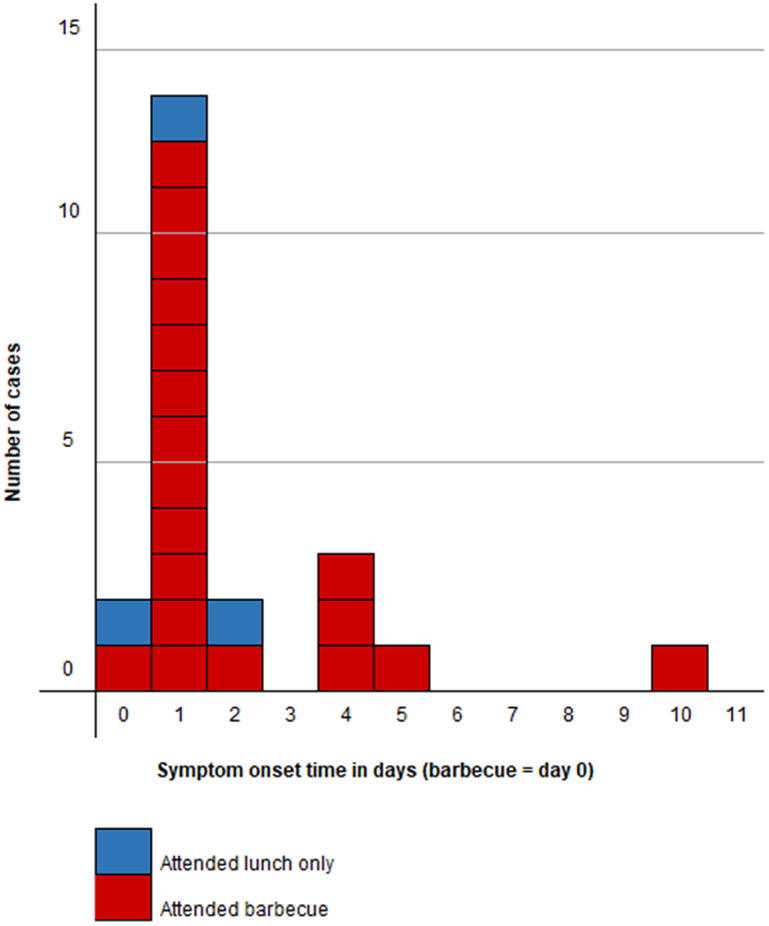
Epidemic curve: day of symptom onset in cases (*n* = 22) by barbecue attendance.

In total, 22 cases were identified in this outbreak (5 confirmed, 17 probable), all of whom were symptomatic, from the same family social network of the Cardiff North African community who attended the barbecue and/or a lunch event (attack rate = 51%). The attack rate of those attending only a lunch event (3/7 = 43%) was the same as those attending only the barbecue (4/9 = 44%). The epidemic curve (Figure 1) is suggestive of a common source, with the majority of cases reporting symptom onset on the day following the barbecue (n = 14). Exact time of onset was known for 14 cases, of whom nine developed symptoms the day after the barbecue (median = 12 h; range = 5 to 12 h). Two further cases developed symptoms a day after this and a further five cases developed symptoms in the following eight days, which is suggestive of person-to-person transmission since some of these cases had attended the barbecue but not become unwell until after the incubation period for salmonellosis.

**Figure 2.**
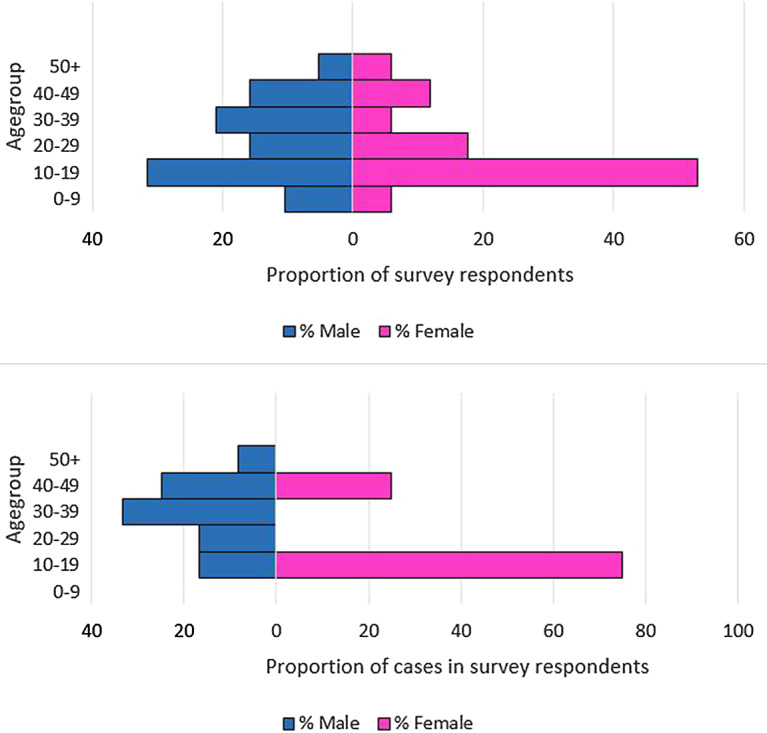
(a) Age–sex profile of all cohort study survey respondents, *n* = 36. (b) Age–sex profile of cases in cohort study survey respondents, *n* = 20.

The discussion with cases and their households about attendance at lunches or the barbecue also gave valuable insight into the wider social network and those visiting Cardiff specifically for these events. We received 36 completed surveys of 43 people in the cohort, of whom 19 were male respondents (Figure 2) and 20 were cases (12 male and 8 female, Figure 2). There were no cases in the 0–9 years age group but men were affected in all other age groups, notably the 30–49 years strata. Females had no cases in all but two strata, but the female 10–19 years age group was the worst affected strata of all respondents. All survey respondents identified were of non-white ethnicity and 30 identified a family connection to the same North African country.

### Cohort study

Of the 36 surveys completed (response rate = 83.7%), 31 lived in Cardiff, and five were visiting Cardiff (from other regions of the UK). These five individuals were staying with friends and family in Cardiff for the Eid Al-Adha celebrations and cited this trip as the only stay outside home in the three days before the barbecue. Only one survey respondent mentioned travel in the three days before the barbecue, where they had visited a local beach with friends for another barbecue. Of all 36 survey responses, there were 20 cases (attack rate = 55.6%; confirmed = 5, probable = 15). The barbecue was attended by 29/36 people (cases = 17), with 20/36 attending both the barbecue and a lunch event (cases = 13). There were seven people attending only a lunch event (cases = 3). All lamb eaten at lunchtime was reported as being stewed for several hours which would have killed salmonella and other enteric bacteria, so was also a less likely vehicle for transmission. However, because some people preparing the lunches reported eating raw offal before the lunch, which could have given rise to cross-contamination such as on salad served at lunchtime, we included these people in our Firth regression analysis. There were 19 male respondents (cases = 12) and 17 female respondents (cases = 8), meaning we were able to interview 91% of the 22 cases in this outbreak. Twelve respondents reported attending emergency health services (A&E) because of their symptoms, six of these were admitted, and four were kept overnight. One patient required intensive care (ICU) to manage an acute electrolyte disturbance and renal function. One respondent noted GI sickness in a barbecue attendee, resident in their household, with symptoms starting before the barbecue. The barbecue food was prepared at home by eight people in the community, five of whom reported doing so in the survey, four of whom were cases and all of whom attended the barbecue. The food was transported, unchilled, to consume together at the evening barbecue, and included marinated uncooked lamb to barbecue, several side dishes, and a number of traditional dishes. These included ‘marara’, made from raw liver, usually cut into strips, washed in lemon juice and vinegar, and marinated in herbs, spices, and peanut butter.

### Univariate analysis

Univariate analysis showed an association between consumption of raw liver at the barbecue and being a case (RR = 2.5, 95% CI: 1.54–4.57, *P* < 0.05) (Table 2). Because the 3 people who reported not eating lamb at the barbecue were not ill, it was not possible to calculate a risk ratio for lamb consumption at the barbecue. The full list of foods reported being consumed at lunch and at the barbecue by those attending the barbecue, and their associated risk ratios from the univariate analysis, are detailed in Table 2. No other significant associations were found. Overall, in the univariate analysis, there were no foods consumed at a lunch event that were significantly associated with being a case.

**Table 2.** Cohort study respondents univariate results (*N* = 36)

Exposure	Exposed			Unexposed					*P*-value^c^
	N	Cases	AR^a^%	N	Cases	AR^a^%	Risk Ratio	95% (CI)^b^	
Lunch (*n* = 27)									
Lamb	12	9	75	17	8	47	1.59	(0.85–3.03)	0.25
Raw kidney	2	2	100	27	15	56	1.8	(0.59–2.68)	0.50
Raw liver	2	2	100	27	15	56	1.8	(0.59–2.68)	0.50
Carrot salad	4	3	75	25	14	56	1.34	(0.51–2.27)	0.62
Other meat	4	2	50	25	15	60	0.83	(0.24–1.69)	1.00
Barbecue (*n* = 29)									
Raw liver	9	9	100	20	8	40	**2.5**	**(1.54–4.57)**	**<0.05**
Lamb^d^	26	17	65	3	0	0	-	(. -.)	**0.06**
Pepsi	9	3	33	20	14	70	0.48	(0.17–1.04)	0.11
Lemonade with yoghurt	10	8	80	19	9	47	1.69	(0.91–3.08)	0.13
Watermelon	16	7	44	13	10	77	0.57	(0.29–1.05)	0.13
Stew	8	3	38	21	14	67	0.56	(0.20–1.19)	0.22
Kisra (flat bread)	8	3	38	21	14	67	0.56	(0.20–1.19)	0.22
Fresh tea	3	3	100	26	14	54	1.86	(0.78–2.82)	0.25
Cooked liver dish	3	3	100	26	14	54	1.86	(0.78–2.82)	0.25
Kunafa (pastry dessert)	15	7	47	14	10	71	0.65	(0.33–1.22)	0.26
Lemonade alone	5	4	80	24	13	54	1.48	(0.65–2.47)	0.37
Mixed green salad	14	7	50	15	10	67	0.75	(0.38–1.40)	0.46
Other drink	12	8	67	17	9	53	1.26	(0.66–2.35)	0.70
Spicy sauce	14	9	64	15	8	53	1.21	(0.64–2.31)	0.71
Mixed carrot salad	3	2	67	26	15	58	1.16	(0.35–2.06)	1.00
Pickled salad	4	2	50	25	15	60	0.83	(0.24–1.69)	1.00
Hummus	4	2	50	25	15	60	0.83	(0.24–1.69)	1.00
Basbousa	10	6	60	19	11	58	1.04	(0.50–1.89)	1.00
Aubergine salad	7	4	57	22	13	59	0.97	(0.40–1.78)	1.00

a Attack rate.

b 95%CI calculated using Wilson score method [10, 11].

c *P*-value calculated using Fisher’s exact method.

d All cases ate lamb at the barbecue; therefore, a RR could not be calculated.

### Firth regression results

Due to the fact that no controls ate raw liver and that all cases ate the lamb at the barbecue, univariate odds ratios could not be calculated, but a significant association was found (Supplementary Table S1). Firth regression was used to analyse these two variables of interest. We also examined the effect of the consumption of spicy sauce in our model because field reports had indicated that some people dipped cooked lamb into the marinade juices from the raw liver dish.

Firth regression revealed that there was no dose–response evident from the consumption of raw liver, (Table 3), but it was evident for lamb; those eating a large portion were 77 times more likely (95%CI 1.2–4,848.8; *P* = 0.04) to be cases than controls. When examining dichotomous variables for lamb and raw liver consumption, lamb was not a significant risk factor but those eating raw liver were 28 times more likely (1.4–546.9; *P* = 0.03) to be cases than controls. Stratification of these three foods revealed that the lamb and spicy sauce were not significant risk factors for illness and confounded the effect of consuming raw liver. Those eating raw liver were 50 times more likely (1.7–1,461.5; *P* = 0.02) to be cases than controls (Table 4). Neither lamb nor spicy sauce improved the fit of our model but raw liver did (LR Chi^2^ = 7.93; *P* = 0.005). We also tested for interaction with sex and age because barbecue attendees reported that males and females sat in separate groups and had differential access to food items. Children were mostly with their mothers, and men had greater access to barbecued lamb. These factors yielded no significant interactions; sex and age did not adjust the likelihood of being a case.

**Table 3.** Firth univariate regression results for food consumed at lunch (*n* = 27) and barbecue (*n* = 29), cohort study survey respondents (*N* = 36)

Food	Cases	Controls	OR	95% CI	*p*-value
Lunch (n = 27)					
Raw liver (small portion)	2	0	4.31	[0.19–98.5]	0.36
Raw kidney (small portion)	2	0	4.31	[0.19–98.1]	0.36
Barbecue (n = 29)					
Raw liver					
any portion	9	0	**27.94**	**[1.43–546.9]**	**0.03**
small portion	3	0	10.29	[0.47–225.9]	0.14
medium portion	2	0	7.35	[0.31–173.1]	0.22
large portion	4	0	13.24	[0.63–279.2]	0.1
BBQ lamb					
any portion	17	9	12.89	[0.60–276.9]	0.1
small portion	9	7	8.87	[0.39–199.6]	0.17
medium portion	3	2	9.8	[0.33–287.4]	0.19
large portion	5	0	**77**	**[1.22–4848.8]**	**0.04**
Spicy sauce					
any portion	16	12	1.52	[0.36–6.4]	0.57
small portion	5	4	1.22	[0.25–6.1]	0.81
medium portion	0	1	0.33	[0.01–9.6]	0.52
large portion	4	0	9	[0.41–198.2]	0.16

**Table 4.** Stratified Firth multivariable regression for food items consumed at barbecue (*N* = 29)

Food (barbecue)	OR	95% CI	*p*-value
BBQ lamb	6.87	[0.28–165.9]	0.24
Raw liver	49.99	**[1.71–1461.5]**	**0.02**
Spicy sauce	0.26	[0.03–2.2]	0.21

### Microbiology and WGS results

Microbiological results were available for ten cases. Nine were PCR-positive for Salmonella Spp., and five were cultured. All five were confirmed as *Salmonella Typhimurium.* Additionally, these five cases were identified as being genetically indistinguishable from one another (0 SNP difference) based on WGS undertaken by the GBRU. There were three cases with more than one pathogen in their stool sample (including two co-infections with Shiga toxin-producing *Escherichia Coli* (STEC) and one with *Campylobacter* Spp.). Nine of these ten cases with microbiological results had eaten raw liver; seven at the barbecue and two at lunch. These latter two also ate raw kidney at lunch. They did not serve raw liver or kidney at lunch but ate it individually. Eight of these ten cases with microbiological results had attended the barbecue and eaten lamb.

### Food microbiology results

Environmental Health Officers retrieved 55 samples of leftover raw lamb, of which 11 were sent for culture testing and genomic sequencing at the GBRU. Samples for other food products used at the barbecue or leftover, including raw sheep’s liver, were unavailable. Sequences obtained from all 11 samples of raw lamb were genetically indistinguishable from one another, and those in the five human isolates from barbecue attendees (0 SNP). These cases had all eaten lamb and raw liver at the barbecue. Two cases also had positive results for STEC infection. Identical genetic sequences to those in this outbreak’s cases were also returned from samples taken from the local butchers and supplying abattoir.

## Discussion

Our results show that attending a lunch event or the barbecue was not a significant predictor for illness in itself, rather the food consumed was. The traditional raw liver dish ‘marara’ was the primary vehicle of transmission in this outbreak. Microbiological evidence suggests that there was widespread contamination of sheep meat consumed at the barbecue, and that cross-contamination with other dishes occurred. However, consumption of infected raw sheep’s liver was likely to have resulted in a high infective dose and therefore an unusually high rate of severe illness. Many cases in this outbreak were severely unwell, with over half (54%, 12/22) of cases seeking emergency hospital care and 27% (6/22) being admitted overnight. Whilst we could not find a statistically significant association between raw liver consumption and hospital attendance, this might have been due to the small sample size. Two cases were also co-infected with STEC, both of whom were hospitalised due to their symptoms. The dual infection might explain the severity of their symptoms, but field interview notes also revealed the high quantity of meat reported in their diets and also cross-contamination issues around food preparation practices.

It was not possible to establish where this contamination occurred in the food chain. Whilst genetically indistinguishable sequences were returned from human, meat, and environmental samples, it is possible that the cooking of the lamb was enough to reduce the infective dose present in the meat at the barbecue, thus affecting people differently through quantity consumed and thoroughness of cooking. It was not possible to test if cross-contamination might have occurred from lunchtime meat preparation in households also preparing food for the evening barbecue, which is a limitation. The people we interviewed as part of the outbreak investigation explained that the barbecue was the principal ‘communal’ meal of the Eid Al-Adha celebrations within this North African family social network in Cardiff. The lunch events were smaller events involving direct family and close friends. However, the Firth regression provides very strong evidence that the raw liver ‘marara’ dish served at the barbecue was the primary transmission vehicle responsible for this outbreak.

The religious practice involving animal slaughter for Eid al-Adha is called ‘Qurbani’ [13]. Consumers undertaking Qurbani in the UK wish to collect their meat and red offal as quickly as possible following slaughter, which means these products might not comply with chilling requirements set out in retained Regulation (EC) 853/2004 [13]. The day of the barbecue was also very hot, and there could have been an amplification effect due to the raw liver and lamb being unchilled for a long period. Our questionnaire responses also indicated that some of the lamb cooked on the barbecue was pink inside and that the marinade from the raw liver was used as a dip for other food and that one person used the plate the raw liver was served on as their plate to eat their meal from. All of this offers multiple opportunities for cross-contamination. The presence of *Salmonella* serovars in sheep livers is well understood [14–18] and it should be assumed that all raw meat and offal is likely to be infected with a variety of pathogens at the time of purchase due to its processing along the food chain.

### Public health messaging implications

Whilst there are many studies focusing on foodborne outbreaks of *Salmonella* infection, the majority of the outbreaks of salmonella which occur in the UK are associated with consumption of poultry and egg products [19]. This outbreak is one of few reported *Salmonella Typhimurium* outbreaks associated with consumption of lamb in the UK [20, 21]. Whilst dishes made from liver are well known for being high-risk foods for campylobacter across the UK, Europe, and the world (poultry liver, such as chicken and duck, [22, 23], and veal liver, Canada [24], there are very few studies describing outbreaks of salmonella linked to offal [25]. We identified one outbreak study from Australia, which identified an association between consumption of both raw and cooked lamb’s liver and *Salmonella Typhimurium* PT 197 in members of the Lebanese community [26]. *Salmonella Typhimurium* has been identified in samples taken from swabs of pig’s livers and tongue in pigs slaughtered for human offal consumption in Portugal, though were not linked to any human cases [27]. Frequently marara also includes boiled sheep’s intestines, though this ingredient was not reported on this occasion.

This common source outbreak acted as a sentinel event, which through further case finding and sequencing led to the identification of samples genetically identical to the outbreak cases, in individuals who were resident of, or visitors to, Cardiff, who had not attended the barbeque or a lunch event.

In addition to the 0-SNP cluster identified, the five sequenced cases in this outbreak were identified as being part of a larger ongoing 5 SNP level cluster; 1.26.136.634.2037.4574.%. Cases in this cluster date back to 2018 and are currently spread across Wales, Scotland, London, and the South West and Midlands of England. At least two of these cases in this wider cluster were in people with occupational contact with sheep farms, suggesting the possibility of a transmission chain with a farm origin. Cases in this cluster have continued to be identified following the close of the barbecue investigation, and investigations continue into the source of these infections and food supply chains.

### Community engagement

Eid al-Adha (‘Festival of Sacrifice’) is one of the most important festivals in the Muslim calendar. The festival remembers the prophet Ibrahim’s willingness to sacrifice his son when God ordered him to. In some countries, Muslims sacrifice a sheep or goat (in Britain, the animal is killed at a slaughter house), and the meat is shared equally between family, friends, and the poor. Eid usually starts with Muslims going to the Mosque for prayer and is a time when they visit family and friends and also give money to charity.

As part of the outbreak response, Public Health Wales sought engagement advice from experts in its service user experience team. Options were considered for engaging the North African community in Cardiff, but also the various communities in Wales, on food safety whilst remaining culturally sensitive and maintaining anonymity. With the support of an engagement coordinator from Cardiff council, an educational session was conducted during the Eid al-Adha festival which included members of the community that consume the traditional ‘marara’ dish and other communities that celebrate Eid al-Adha. Key food safety and hygiene messages were presented followed by an open conversation. The session received positive verbal feedback from the participants. Learning from this engagement helped Public Health Wales develop a universal response (Box 1), available in several languages, to food safety that is now programmed into annual communications for World Food Safety Day in June for the whole population and also ahead of specific festivals and events such as Eid al-Adha. The approach consisted of two phases:

- *Phase one*: general food safety messages shared via social media on World Food Safety Day in June 2022, available in several languages (Supplementary Figure S1).
- *Phase two*: Food safety infographics tailored for the Eid al-Adha festival in July 2022. Infographics were shared via Public Health Wales’s social media platforms, external partners, and the WhatsApp group established for sharing pandemic information with communities.

Box 1. Lessons learnt from community engagement

1. Setting clear and realistic engagement objectives from the project outset
2. Ensure resources are adequate to deliver on objectives and recommendations
3. Allow sufficient time for translation of materials
4. Ensure partnership working with other statutory partners and align the engagement where possible
5. Establish contact early with communities and partners to enable maximum participation and publicity
6. Ensure networks are maintained regularly so there is willingness to support investigations when needed

## Conclusions

Whilst the consumption of raw or undercooked meats is associated with salmonellosis outbreaks in Europe [6, 7], this outbreak in a minority ethnic community in South Wales identified a new vehicle of interest, a traditional raw offal dish. Future outbreak investigations, particularly where cultural events are associated with particular foods, should consider dishes beyond those on routine questionnaires to those which may be relevant to the community in question.

Consumption of raw and undercooked meat and offal continues to be an ongoing public health risk. Improving awareness and proactive messaging, especially to communities who consume raw/undercooked meat or offal either regularly, or during specific religious or cultural events, may help reduce this risk.

## Supporting information

Adamson et al. supplementary material 1 Adamson et al. supplementary material

Adamson et al. supplementary material 2 Adamson et al. supplementary material

Adamson et al. supplementary material 3 Adamson et al. supplementary material

## Data Availability

The data used in this investigation contain personal identifiable information. Anonymised information, including that contained in the supplementary information, required to reproduce these results is available from the corresponding author on reasonable request.

## Supplementary material

The supplementary material for this article can be found at https://doi.org/10.1017/S0950268823001887.
