# Supplementary figures and images for: An outbreak of Salmonella Typhimurium associated with the consumption of raw liver at an Eid al-Adha celebration in Wales (UK), July 2021
Source: Epidemiol Infect. 2023 Nov 30;152:e6. doi: 10.1017/S0950268823001887 (PMC10789983; doi:10.1017/S0950268823001887)

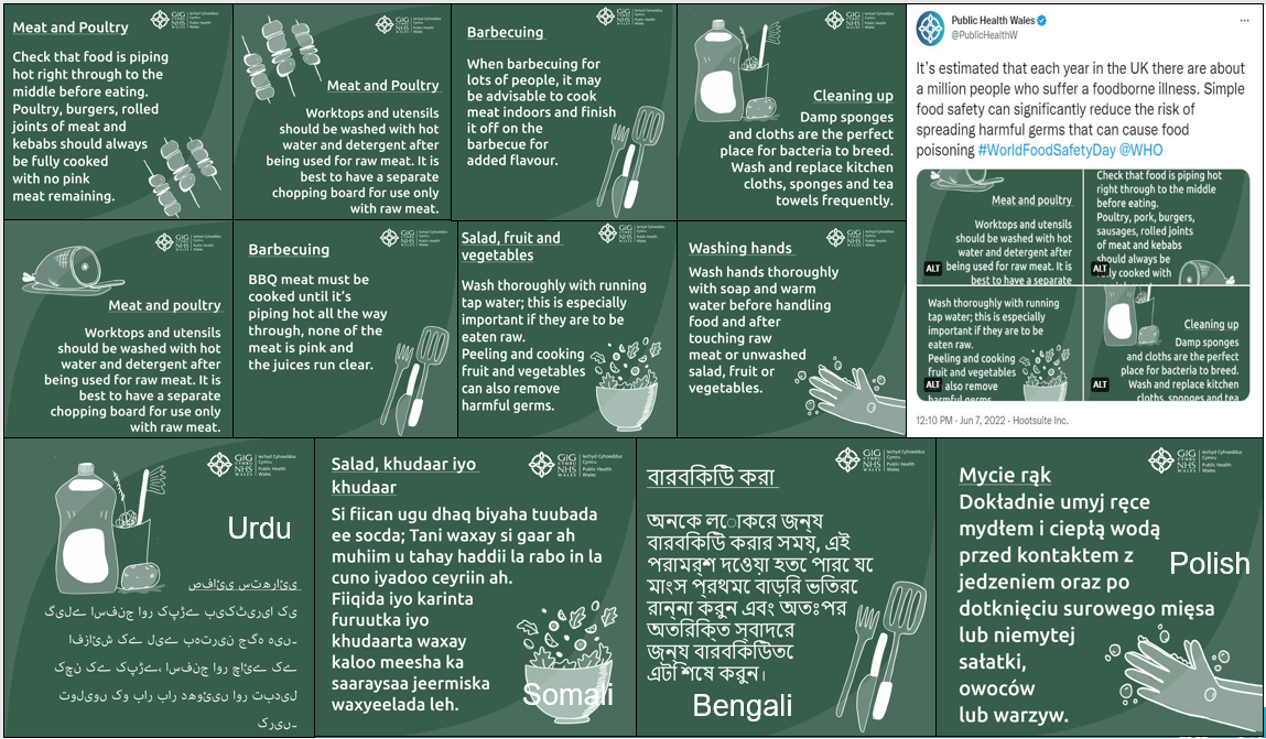

Supplement: Adamson et al. supplementary material 1 — Adamson et al. supplementary material [file S0950268823001887sup001.png]
